# Synthesis and crystal structure of *N*-(4-chloro­phen­yl)-5,7-dimethyl-1,2,4-triazolo[1,5-*a*]pyrimidin-2-amine

**DOI:** 10.1107/S2056989016019629

**Published:** 2017-01-01

**Authors:** Hlib Repich, Svitlana Orysyk, Pavlo Savytskyi, Vasyl Pekhnyo

**Affiliations:** a Vernadsky Institute of General and Inorganic Chemistry of the Ukrainian National Academy of Sciences, Palladin av. 32/34, 03142 Kyiv, Ukraine; b The Institute of Molecular Biology and Genetics of the Ukrainian National Academy of Sciences, Zabolotnogo Str. 150, 03680 Kyiv, Ukraine

**Keywords:** crystal structure, thio­semicarbazide cyclization, triazolo­pyrimidines, Dimroth rearrangement

## Abstract

The title compound, C_13_H_12_ClN_5_, was synthesized by the cyclization of 1-(4,6-di­methyl­pyrimidin-2-yl)-4-phenyl­thio­semicarbazide in the presence of Ni(NO_3_)_2_. The mol­ecular structure of the compound is essentially planar. In the crystal, mol­ecules form dimers *via* pairs of N—H⋯N hydrogen bonds between the H atom of the exocyclic amino group and the N atom at the 4-position of the triazole ring. The resulting dimers are packed into layers which are connected by π-stacking inter­actions between the aromatic systems of the pyrimidine and benzene nuclei, and between the triazole cores.

## Abstract 2

The title compound, *N*-(4-chloro­phen­yl)-5,7-dimethyl-1,2,4-triazolo[1,5-*a*]pyrimidin-2-amine, was synthesized by cyclization of 1-(4,6-di­methyl­pyrimidin-2-yl)-4-phenyl­thio­semicarbazide in the presence of Ni(NO_3_)_2_. In the crystal, mol­ecules form inversion dimers *via* pairs of N—H⋯N hydrogen bonds, which are packed into layers by π-stacking inter­actions between the aromatic systems of neighbouring mol­ecules.

## Chemical context   

**Figure 1:**
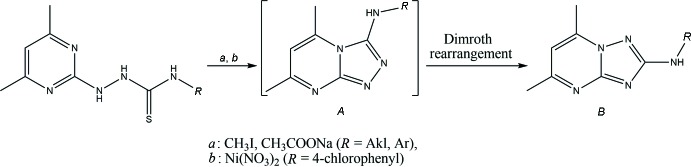
Scheme showing the formation of related compounds (*a*) according to the literature and (*b*) in the present work.

It is well known that thermal cyclization of 1-(pyrymidin-2-yl)thio­semicarbazides leads to the formation of mercapto derivatives of triazolo­pyrimidine (Babichev & Kovtunenko, 1977 ▸; Kottke & Kuhmshtedt, 1978 ▸). In contrast to this, it has been shown that analogous substrates can be converted into the corresponding 2-*R*-amino-5,7-dimeth­yl[1,2,4]triazolo[1,5-*a*]pyrimidines by cyclization in the presence of methyl iodide and sodium acetate in boiling ethanol solution. Such processes undergo alcylation of a sulfur atom with the formation of the *S*-methyl derivative, which then undergoes intra­molecular cyclization with elimination of a methane­thiol mol­ecule and the formation of the unstable inter­mediate *A*. The subsequent Dimroth rearrangement of inter­mediate *A* gives the final product *B* (Fig. 1 ▸) (Vas’kevich *et al.*, 2006 ▸). In the present work we show that an analogous cyclization followed by Dimroth rearrangement can proceed in mild conditions in the presence of Ni^2+^ ions (Fig. 1 ▸).

## Structural commentary   

The mol­ecular structure of the title compound is almost planar. The mol­ecule consists of two flat fragments: the [1,2,4]triazolo[1,5-*a*]pyrimidine moiety, and the 4-chloro­phenyl group. The mean deviation from the N1/C2/C3/C4/N2/C6/N3/C7/N4 plane is 0.010 Å while that from the C8–C13 plane is 0.006 Å. The dihedral angle between these planes is 6.23 (5)°. The sum of the C7—N5—C8, C7—N5—H1 and C8—N5—H1 angles is 359.86°, indicating *sp* ^2^ hybridization of atom N5.
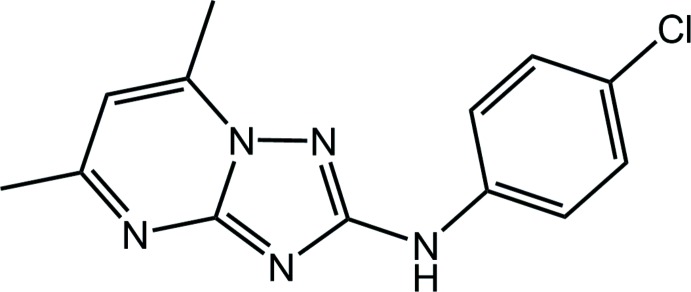


## Supra­molecular features   

**Figure 2:**
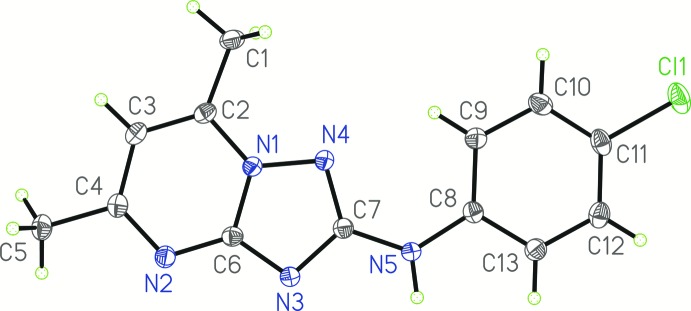
The mol­ecular structure of the title compound. Displacement ellipsoids are drawn at the 50% probability level.

**Figure 3:**
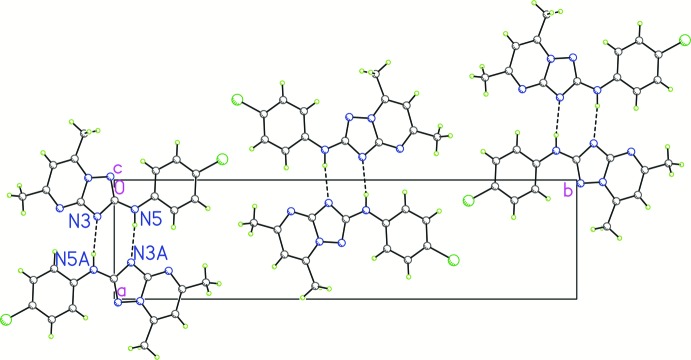
Packing diagram of the title compound with N—H⋯N hydrogen bonds shown as dashed lines. The projection is shown along [001] and the atoms labelled with suffix *A* are related by an inversion centre (symmetry code 1 − *x*, −*y*, 2 − *z*).

In the crystal, mol­ecules form inversion dimers *via* pairs of N5—H1⋯N3^i^ hydrogen bonds (Table 1 ▸, Fig. 2 ▸). The resulting dimers are packed into layers parallel to the *bc* plane. These layers are connected by π-stacking inter­actions between the aromatic systems of the pyrimidine and benzene rings, and between triazole cores (Figs. 3 ▸ and 4 ▸). The centroid–centroid distance between the benzene ring of the 4-chloro­phenyl group (C8–C13) and the pyrimidine ring (N1/C2/C3/C4/N2/C6) of symmetry-related mol­ecules is 3.513 (1) Å. These overlapping rings have a slip angle of 16.3°. The centroid–centroid distance between five-membered (N1/N4/C7/N3/C6) triazole rings is 3.824 (1) Å with a slip angle of 29.0°.

In general, the crystal structure of the title compound is very similar to that of 5,7-dimethyl-2-phenyl­amino-1,2,4-triazolo[1,5-*a*]pyrimidine (Vas’kevich *et al.*, 2006 ▸).

## Synthesis and crystallization   

A warm solution of Ni(NO_3_)_2_ (0.0364 g, 0.125 mmol in 15 ml of ethanol) was added dropwise under vigorous stirring to a warm solution of 1-(4,6-di­methyl­pyrimidin-2-yl)-4-phenyl­thio­semicarbazide (0.0767 g, 0.25 mmol in 20 ml of ethanol), prepared according to a known procedure (Vas’kevich *et al.*, 2006 ▸). An orange precipitate of the Ni^2+^ complex (*M*:*L* = 1:2) was formed. The resulting mixture was left for a few days. Detailed analysis of the obtained compound showed the presence of a significant amount of colourless plate-shaped crystals of the title compound, which were used for X-ray analysis.

## Refinement   

Crystal data, data collection and structure refinement details are summarized in Table 2 ▸. All H atoms bonded to C atoms were placed in geometrically idealized positions according to hybridization and constrained to ride on their parent C atoms, with C—H bonds for the aromatic rings and methyl groups of 0.95 and 0.98 Å, respectively, with *U*_iso_(H_aromatic_) = 1.2*U*_eq_(C) and *U*_iso_(H_meth­yl_) = 1.5*U*_eq_(C). The methyl groups were allowed to rotate freely about the C—C bonds. The H atom bonded to the N atom was located in a difference map and refined without any restraints.

## Supplementary Material

Crystal structure: contains datablock(s) I. DOI: 10.1107/S2056989016019629/lh5830sup1.cif

Structure factors: contains datablock(s) I. DOI: 10.1107/S2056989016019629/lh5830Isup2.hkl

Supporting information file. DOI: 10.1107/S2056989016019629/lh5830Isup3.cml

CCDC reference: 1521445

Additional supporting information: crystallographic information; 3D view; checkCIF report

## Figures and Tables

**Figure 4:**
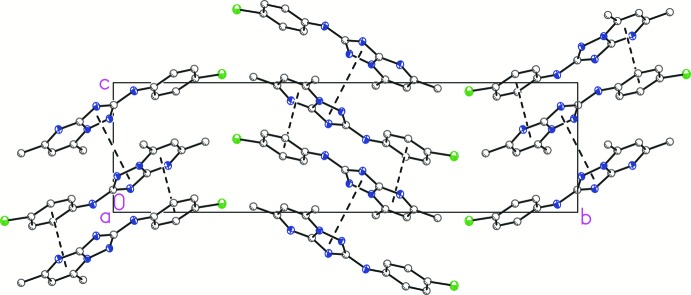
Packing diagram of the title compound with π–π inter­actions between aromatic systems represented by dashed lines. The projection is shown along [100]. H atoms have been omitted for clarity.

**Table 1:** Hydrogen-bond geometry (Å, °)

*D*—H⋯*A*	*D*—H	H⋯*A*	*D*⋯*A*	*D*—H⋯*A*
N5—H1⋯N3^i^	0.870 (18)	2.109 (18)	2.9748 (14)	173.5 (16)

Symmetry code: (i) 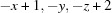.

**Table 2:** Experimental details

Crystal data	
Chemical formula	C_13_H_12_ClN_5_
*M*_r_	273.73
Crystal system, space group	Monoclinic, *P*2_1_/*n*
Temperature (K)	100
*a*, *b*, *c* (Å)	7.0640 (1), 25.2362 (4), 7.6494 (1)
β (°)	113.243 (1)
*V* (Å^3^)	1252.97 (3)
*Z*	4
Radiation type	Mo *K*α
μ (mm^−1^)	0.30
Crystal size (mm)	0.40 × 0.30 × 0.05

Data collection	
Diffractometer	Bruker APEXII CCD
Absorption correction	Multi-scan (*SADABS*; Bruker, 2001 ▸)
*T*_min_, *T*_max_	0.874, 0.985
No. of measured, independent and observed [*I* > 2σ(*I*)] reflections	11572, 3837, 3347
*R*_int_	0.018
(sin θ/λ)_max_ (Å^−1^)	0.716

Refinement	
*R*[*F* ^2^ > 2σ(*F* ^2^)], *wR*(*F* ^2^), *S*	0.039, 0.100, 1.04
No. of reflections	3837
No. of parameters	178
H-atom treatment	H atoms treated by a mixture of independent and constrained refinement
Δρ_max_, Δρ_min_ (e Å^−3^)	0.47, −0.33

Computer programs: *APEX2* and *SAINT* (Bruker, 2007 ▸), *SHELXS97* and *SHELXTL* (Sheldrick, 2008 ▸), *SHELXL2014* (Sheldrick, 2015 ▸) and *publCIF* (Westrip, 2010 ▸).

## supplementary crystallographic information

### Crystal data

**Table:**

C_13_H_12_ClN_5_	*F*(000) = 568
*M**_r_* = 273.73	*D*_x_ = 1.451 Mg m^−^^3^
Monoclinic, *P*2_1_/*n*	Mo *K*α radiation, λ = 0.71073 Å
*a* = 7.0640 (1) Å	Cell parameters from 5527 reflections
*b* = 25.2362 (4) Å	θ = 3.0–30.5°
*c* = 7.6494 (1) Å	µ = 0.30 mm^−^^1^
β = 113.243 (1)°	*T* = 100 K
*V* = 1252.97 (3) Å^3^	Plate, colorless
*Z* = 4	0.40 × 0.30 × 0.05 mm

### Data collection

**Table:**

Bruker APEXII CCD diffractometer	3347 reflections with *I* > 2σ(*I*)
Radiation source: sealed tube	*R*_int_ = 0.018
φ and ω scans	θ_max_ = 30.6°, θ_min_ = 1.6°
Absorption correction: multi-scan (SADABS; Bruker, 2001)	*h* = −10→8
*T*_min_ = 0.874, *T*_max_ = 0.985	*k* = −36→33
11572 measured reflections	*l* = −6→10
3837 independent reflections	

### Refinement

**Table:**

Refinement on *F*^2^	Primary atom site location: structure-invariant direct methods
Least-squares matrix: full	Secondary atom site location: difference Fourier map
*R*[*F*^2^ > 2σ(*F*^2^)] = 0.039	Hydrogen site location: mixed
*wR*(*F*^2^) = 0.100	H atoms treated by a mixture of independent and constrained refinement
*S* = 1.04	*w* = 1/[σ^2^(*F*_o_^2^) + (0.047*P*)^2^ + 0.6408*P*] where *P* = (*F*_o_^2^ + 2*F*_c_^2^)/3
3837 reflections	(Δ/σ)_max_ = 0.001
178 parameters	Δρ_max_ = 0.47 e Å^−^^3^
0 restraints	Δρ_min_ = −0.33 e Å^−^^3^

### Special details

**Table:**

Geometry. All esds (except the esd in the dihedral angle between two l.s. planes) are estimated using the full covariance matrix. The cell esds are taken into account individually in the estimation of esds in distances, angles and torsion angles; correlations between esds in cell parameters are only used when they are defined by crystal symmetry. An approximate (isotropic) treatment of cell esds is used for estimating esds involving l.s. planes.
Refinement. Refinement of *F*^2^ against ALL reflections. The weighted *R*-factor *wR* and goodness of fit *S* are based on *F*^2^, conventional *R*-factors *R* are based on *F*, with *F* set to zero for negative *F*^2^. The threshold expression of *F*^2^ > σ(*F*^2^) is used only for calculating *R*-factors(gt) *etc*. and is not relevant to the choice of reflections for refinement. *R*-factors based on *F*^2^ are statistically about twice as large as those based on *F*, and *R*- factors based on ALL data will be even larger.

### Fractional atomic coordinates and isotropic or equivalent isotropic displacement parameters (Å^2^)

**Table:**

	*x*	*y*	*z*	*U*_iso_*/*U*_eq_	
C1	−0.39001 (18)	−0.06515 (5)	0.47760 (19)	0.0212 (2)	
H1A	−0.4039	−0.0542	0.5949	0.032*	
H1B	−0.4903	−0.0931	0.4153	0.032*	
H1C	−0.4154	−0.0347	0.3919	0.032*	
C2	−0.17835 (18)	−0.08555 (5)	0.52419 (16)	0.0163 (2)	
C3	−0.12544 (19)	−0.13199 (5)	0.46223 (17)	0.0176 (2)	
H3	−0.2303	−0.1544	0.3786	0.021*	
C4	0.08323 (19)	−0.14699 (5)	0.52120 (17)	0.0171 (2)	
C5	0.1395 (2)	−0.19827 (5)	0.45558 (19)	0.0212 (2)	
H5C	0.2813	−0.1962	0.4637	0.032*	
H5B	0.0457	−0.2050	0.3235	0.032*	
H5A	0.1285	−0.2272	0.5366	0.032*	
C6	0.18434 (17)	−0.07241 (4)	0.69865 (16)	0.0154 (2)	
C7	0.17125 (17)	0.00081 (4)	0.82697 (16)	0.0152 (2)	
C8	0.13730 (18)	0.08741 (4)	0.96620 (16)	0.0156 (2)	
C9	−0.07753 (18)	0.08999 (5)	0.89705 (17)	0.0179 (2)	
H9	−0.1590	0.0613	0.8262	0.022*	
C10	−0.17128 (19)	0.13482 (5)	0.93266 (18)	0.0203 (2)	
H10	−0.3172	0.1366	0.8871	0.024*	
C11	−0.0532 (2)	0.17674 (5)	1.03401 (17)	0.0199 (2)	
C12	0.1601 (2)	0.17448 (5)	1.10631 (18)	0.0202 (2)	
H12	0.2404	0.2031	1.1785	0.024*	
C13	0.25465 (19)	0.12990 (5)	1.07201 (17)	0.0184 (2)	
H13	0.4008	0.1281	1.1208	0.022*	
Cl1	−0.17415 (6)	0.23384 (2)	1.06639 (5)	0.02889 (10)	
H1	0.378 (3)	0.0447 (7)	1.006 (2)	0.023 (4)*	
N1	−0.01741 (15)	−0.05610 (4)	0.64112 (14)	0.01484 (19)	
N3	0.30697 (15)	−0.03648 (4)	0.81768 (15)	0.01627 (19)	
N5	0.24606 (16)	0.04442 (4)	0.93749 (15)	0.0171 (2)	
N4	−0.02837 (15)	−0.00828 (4)	0.72245 (14)	0.01575 (19)	
N2	0.23744 (16)	−0.11766 (4)	0.63896 (15)	0.0173 (2)	

### Atomic displacement parameters (Å^2^)

**Table:**

	*U*^11^	*U*^22^	*U*^33^	*U*^12^	*U*^13^	*U*^23^
C1	0.0139 (5)	0.0218 (6)	0.0243 (6)	−0.0013 (4)	0.0036 (5)	0.0008 (5)
C2	0.0154 (5)	0.0171 (5)	0.0145 (5)	−0.0025 (4)	0.0038 (4)	0.0020 (4)
C3	0.0183 (5)	0.0169 (5)	0.0161 (5)	−0.0041 (4)	0.0050 (4)	−0.0006 (4)
C4	0.0210 (5)	0.0155 (5)	0.0163 (5)	−0.0013 (4)	0.0090 (4)	0.0000 (4)
C5	0.0253 (6)	0.0178 (5)	0.0220 (6)	−0.0013 (4)	0.0109 (5)	−0.0046 (4)
C6	0.0144 (5)	0.0160 (5)	0.0161 (5)	−0.0008 (4)	0.0064 (4)	0.0012 (4)
C7	0.0153 (5)	0.0147 (5)	0.0156 (5)	−0.0002 (4)	0.0060 (4)	0.0006 (4)
C8	0.0176 (5)	0.0146 (5)	0.0144 (5)	0.0018 (4)	0.0063 (4)	0.0017 (4)
C9	0.0168 (5)	0.0180 (5)	0.0178 (5)	0.0006 (4)	0.0056 (4)	−0.0002 (4)
C10	0.0186 (5)	0.0223 (6)	0.0192 (6)	0.0053 (4)	0.0068 (5)	0.0024 (4)
C11	0.0271 (6)	0.0167 (5)	0.0166 (5)	0.0073 (4)	0.0092 (5)	0.0026 (4)
C12	0.0264 (6)	0.0156 (5)	0.0174 (5)	0.0003 (4)	0.0074 (5)	0.0006 (4)
C13	0.0189 (5)	0.0162 (5)	0.0184 (5)	0.0002 (4)	0.0056 (4)	0.0006 (4)
Cl1	0.03686 (19)	0.02176 (16)	0.02714 (18)	0.01310 (12)	0.01167 (14)	0.00019 (12)
N1	0.0142 (4)	0.0138 (4)	0.0160 (5)	−0.0005 (3)	0.0053 (4)	−0.0002 (3)
N3	0.0140 (4)	0.0153 (4)	0.0190 (5)	−0.0003 (3)	0.0061 (4)	−0.0019 (4)
N5	0.0128 (4)	0.0158 (4)	0.0203 (5)	0.0002 (3)	0.0040 (4)	−0.0031 (4)
N4	0.0149 (4)	0.0134 (4)	0.0175 (5)	−0.0003 (3)	0.0050 (4)	−0.0013 (3)
N2	0.0177 (5)	0.0161 (4)	0.0192 (5)	−0.0011 (4)	0.0084 (4)	−0.0015 (4)

### Geometric parameters (Å, º)

**Table:**

C1—C2	1.4857 (16)	C7—N5	1.3612 (15)
C1—H1A	0.9800	C7—N3	1.3651 (14)
C1—H1B	0.9800	C8—N5	1.3960 (14)
C1—H1C	0.9800	C8—C9	1.3977 (16)
C2—N1	1.3574 (15)	C8—C13	1.4008 (16)
C2—C3	1.3702 (16)	C9—C10	1.3911 (16)
C3—C4	1.4124 (17)	C9—H9	0.9500
C3—H3	0.9500	C10—C11	1.3805 (18)
C4—N2	1.3309 (15)	C10—H10	0.9500
C4—C5	1.4976 (16)	C11—C12	1.3859 (18)
C5—H5C	0.9800	C11—Cl1	1.7423 (12)
C5—H5B	0.9800	C12—C13	1.3855 (16)
C5—H5A	0.9800	C12—H12	0.9500
C6—N3	1.3338 (15)	C13—H13	0.9500
C6—N2	1.3374 (15)	N1—N4	1.3737 (13)
C6—N1	1.3781 (15)	N5—H1	0.870 (18)
C7—N4	1.3381 (15)		
			
C2—C1—H1A	109.5	N5—C8—C9	124.01 (11)
C2—C1—H1B	109.5	N5—C8—C13	116.67 (11)
H1A—C1—H1B	109.5	C9—C8—C13	119.32 (11)
C2—C1—H1C	109.5	C10—C9—C8	119.56 (11)
H1A—C1—H1C	109.5	C10—C9—H9	120.2
H1B—C1—H1C	109.5	C8—C9—H9	120.2
N1—C2—C3	115.11 (10)	C11—C10—C9	120.28 (11)
N1—C2—C1	118.05 (11)	C11—C10—H10	119.9
C3—C2—C1	126.84 (11)	C9—C10—H10	119.9
C2—C3—C4	120.77 (11)	C10—C11—C12	120.89 (11)
C2—C3—H3	119.6	C10—C11—Cl1	119.47 (10)
C4—C3—H3	119.6	C12—C11—Cl1	119.62 (10)
N2—C4—C3	122.65 (11)	C11—C12—C13	119.16 (11)
N2—C4—C5	116.91 (11)	C11—C12—H12	120.4
C3—C4—C5	120.42 (11)	C13—C12—H12	120.4
C4—C5—H5C	109.5	C12—C13—C8	120.76 (11)
C4—C5—H5B	109.5	C12—C13—H13	119.6
H5C—C5—H5B	109.5	C8—C13—H13	119.6
C4—C5—H5A	109.5	C2—N1—N4	126.67 (10)
H5C—C5—H5A	109.5	C2—N1—C6	122.58 (10)
H5B—C5—H5A	109.5	N4—N1—C6	110.72 (9)
N3—C6—N2	128.28 (11)	C6—N3—C7	102.94 (9)
N3—C6—N1	109.03 (10)	C7—N5—C8	128.56 (10)
N2—C6—N1	122.69 (10)	C7—N5—H1	116.2 (11)
N4—C7—N5	124.68 (10)	C8—N5—H1	115.1 (11)
N4—C7—N3	116.54 (10)	C7—N4—N1	100.77 (9)
N5—C7—N3	118.77 (10)	C4—N2—C6	116.17 (10)
			
N1—C2—C3—C4	0.80 (16)	N2—C6—N1—C2	2.19 (17)
C1—C2—C3—C4	−178.75 (11)	N3—C6—N1—N4	0.47 (13)
C2—C3—C4—N2	0.44 (18)	N2—C6—N1—N4	−179.62 (10)
C2—C3—C4—C5	178.90 (11)	N2—C6—N3—C7	−179.97 (12)
N5—C8—C9—C10	−179.67 (11)	N1—C6—N3—C7	−0.07 (12)
C13—C8—C9—C10	0.64 (17)	N4—C7—N3—C6	−0.38 (14)
C8—C9—C10—C11	0.64 (18)	N5—C7—N3—C6	−179.42 (10)
C9—C10—C11—C12	−1.73 (19)	N4—C7—N5—C8	0.2 (2)
C9—C10—C11—Cl1	176.72 (9)	N3—C7—N5—C8	179.17 (11)
C10—C11—C12—C13	1.50 (18)	C9—C8—N5—C7	6.89 (19)
Cl1—C11—C12—C13	−176.95 (9)	C13—C8—N5—C7	−173.41 (11)
C11—C12—C13—C8	−0.19 (18)	N5—C7—N4—N1	179.62 (11)
N5—C8—C13—C12	179.42 (11)	N3—C7—N4—N1	0.64 (13)
C9—C8—C13—C12	−0.87 (18)	C2—N1—N4—C7	177.46 (11)
C3—C2—N1—N4	−179.95 (10)	C6—N1—N4—C7	−0.64 (12)
C1—C2—N1—N4	−0.35 (17)	C3—C4—N2—C6	−0.44 (17)
C3—C2—N1—C6	−2.06 (16)	C5—C4—N2—C6	−178.96 (10)
C1—C2—N1—C6	177.54 (10)	N3—C6—N2—C4	179.07 (11)
N3—C6—N1—C2	−177.72 (10)	N1—C6—N2—C4	−0.82 (16)

### Hydrogen-bond geometry (Å, º)

**Table:**

*D*—H···*A*	*D*—H	H···*A*	*D*···*A*	*D*—H···*A*
N5—H1···N3^i^	0.870 (18)	2.109 (18)	2.9748 (14)	173.5 (16)

Symmetry code: (i) −*x*+1, −*y*, −*z*+2.
